# Kilimanjaro Abruzzo expedition: effects of high-altitude trekking on anthropometric, cardiovascular and blood biochemical parameters

**DOI:** 10.1007/s11332-015-0235-z

**Published:** 2015-08-21

**Authors:** Vittore Verratti, S. Falone, C. Doria, T. Pietrangelo, C. Di Giulio

**Affiliations:** Department of Neurosciences, Imaging and Clinical Sciences, “G. d’Annunzio” University, Chieti, Italy; Department of Life, Health and Environmental Sciences, University of L’Aquila, L’Aquila, Italy

**Keywords:** Physical exercise, Altitude, Hypoxia, Human adaptations

## Abstract

The effect of the combination of trekking and balanced appropriated diet were studied in mountaineers who spent 6 days at an altitude ranging from 900 to 5895 m above sea level (a.s.l.), during the Kilimanjaro Abruzzo Expedition. This study explored whether anthropometric, cardiovascular and blood biochemical parameters were significantly changed by a regular trekking performed at high altitude, with reduced oxygen levels, together with a macronutrient-containing balanced diet (total daily caloric intake: 3000–3500 Kcals). In consideration of the short period of high-altitude exposure, high-altitude exercise appeared to provide beneficial and rapid effects on the lipid profile and to modulate cardiovascular functions. These effects rely on both high-altitude hypoxia and physical activity. The most interesting observation is that even just a few days of high-altitude exercise, along with a balanced diet, was able to improve plasma lipid profiles.

## Introduction

Recent studies underscore the role of oxygen availability in different aspects of medical sciences, in physiology and pathophysiology but also in studies of human pathology and in the study of tumors.

Specifically, considering only aspects of human adaptations, several authors have studied the influence of exercise at high altitude. A prolonged and active sojourn in high-altitude hypoxic environments may decrease body weight, body mass index (BMI), waist circumference, without affecting significantly the lean body mass [1–4] which, however, tends to be reduced in response to prolonged exposure to high altitude [5].

Exposure to high altitudes is known to change cardiopulmonary functions. High-altitude hypoxic environment triggers cardiovascular and ventilatory responses which are related to alterations of saturation of peripheral oxygen (SpO_2_), and this depends on the pattern and intensity of the hypoxic exposure. Many days of hypoxia cause a hypoxic ventilatory acclimatization, through which humans undergo major adaptations to high altitude [6]. Cardiac output was reported to be improved immediately after exposure to hypoxia; heart rate has been also shown to increase, mainly due to catecholamine-based stimulations [7]. It has been reported that during submaximal exercise on simulated hypoxia, the hypoxemic stimulation of chemoreceptors induces central physiological responses such as tachycardia and hyperventilation [8]. In this study, we observed an increased heart rate at rest and such a response to hypoxia is temporary. Indeed, after a few days of acclimatization, cardiac output returned to base-line values, though heart rate remained above pre-exposure conditions [9].

During a short-term altitude acclimatization, blood pressure slightly increases, through the activation of arterial chemoreceptor of the sympathetic nervous system [10]. However, on the other hand, some authors suggest that permanent residents at high altitudes exhibit a reduced blood pressure [10, 11]. Upon more prolonged (several days) hypoxic exposures, research has shown a progressive increase in mean arterial blood pressure and diastolic blood pressure, along with elevated plasma concentrations and urinary excretion rates of norepinephrine [10, 12]. Several factors could influence cardiocirculatory behavior. Hypoxia represents the main factor for arterial blood pressure increase. Chronic hypoxia may exert a tonic effect on peripheral chemoreceptors by the increased arterial blood pressure in normal subjects [13] and, for example, during wakefulness [14].

Correlations among exercise, anthropometric, cardiovascular and blood biochemical parameters in high-altitude environments are difficult to be defined due to the heterogeneity of the factors that affect such parameters. In a finding that is consistent with this phenomenon, it is often difficult to draw conclusions from published studies dealing with exercise at high altitude. Duration, intensity, temperature, power, fatigue, age, gender and clinical conditions of the subjects under investigation are some of the many variables confounding the interpretation of the phenomena related to physical exercise at high altitude. As a result of this, one can conclude that negative effects of physical activity at high altitude are predominant on the positive effects. However, exercise, together with a healthy diet, balanced for energy needs (calories) and nutrients (proteins, carbohydrates, fats, vitamins, minerals), represents the best non-pharmacological intervention to preserve good health status. Generally, the mountain ascending during trekking and leisure activities entails a submaximal exercise. Recently, it has been reported that an intensity of exercise ranging from 20 to 40 % of maximal aerobic power performed at 3000–4800 m of altitude does not entail ventilatory or cardiac responses on male young volunteers. In fact, both ventilatory and cardiac responses to hypoxia at submaximal exercise are independent of altitude and exercise intensity and in particular, the peripheral extraction of oxygen is not a limiting factor of submaximal exercise in hypoxic-simulated altitude [8].

In this study, we planned to evaluate the combined effects of high altitude, physical activity and dietary control in male mountaineers who climbed Mount Kilimanjaro (5895 m a.s.l.) and spent 6 days at altitudes ranging from 900 to 5895 m a.s.l. on the cardiovascular and blood biochemical parameters. We believe that such a short and middle intensity exercise can provide an optimal condition to assess whether exercise in altitude can be beneficial. This condition is different from the typical Himalayan expedition lasting several weeks and implying a sojourn for many days at an altitude of over 5000 m a.s.l., and also different from the classical ascent of a high top of the Alps where the mountaineers are engaged only for 1–2 days with a very limited period spent above 3000 m a.s.l.

To give special emphasis on the effect for health, we evaluated the variations of the health status using standardized diagnostic tools and routine laboratory analyses for cardiovascular, metabolic and inflammatory parameters.

## Methods

The Laboratory of Functional Evaluation of the Department of Neuroscience, Imaging and Clinical Sciences, “G. D’Annunzio” University, Chieti-Pescara Italy, in cooperation with the Laboratory of Clinical Pathology of the Teramo Hospital, carried out an experimental project “Kilimanjaro Abruzzo Expedition” which was aimed at investigating the physiological responses activated by high altitude and exercise, in association with a balanced diet. The research was approved by the Bioethical Committee of the University of Chieti and carried out in accordance with the Declaration of Helsinki (as revised in Edinburgh, in 2000). The participants provided written informed consent before participating in the study.

### Participants, study protocol, measurements

**Fig. 1:**
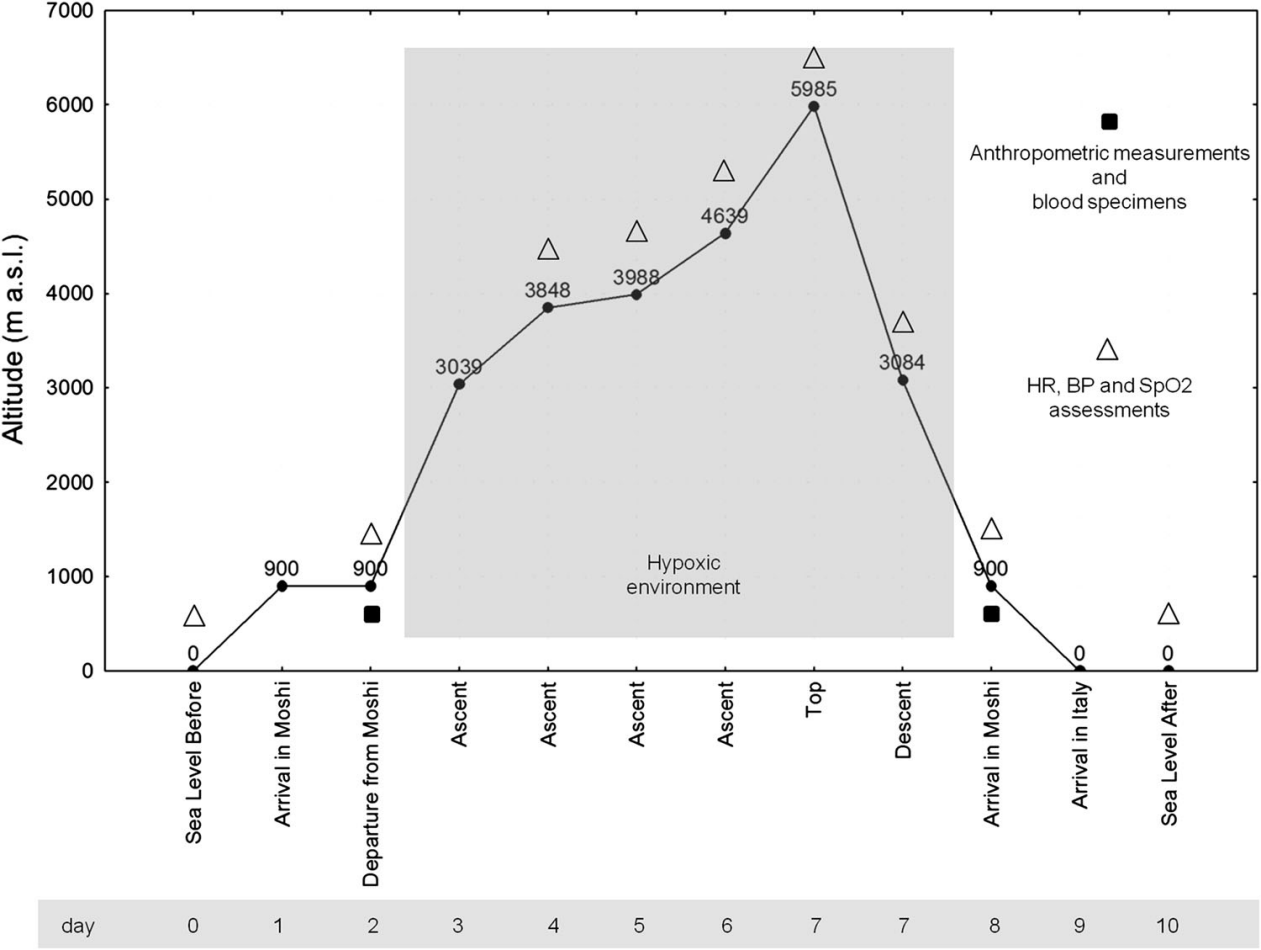
Kilimanjaro Abruzzo Expedition altimetric profile

All measurements were made on twelve, male, non-acclimatized mountain climbers (age 45 ± 9 years old), who spent 6 days at an altitude ranging from 900 m a.s.l. (in Moshi, which is situated on the lower slopes of Mt. Kilimanjaro, Northern Tanzania) to 5895 m a.s.l. (Uhuru Peak, Kilimanjaro summit), during the Kilimanjaro Abruzzo Expedition, as reported in the altimetric profile (Fig. 1).

During this period, the mountaineers were engaged everyday in regular trekking activities and received meals prepared by local porters with a balanced diet (see Table 1) of macronutrients (carbohydrates 50 %, fats 30 %, proteins 20 %), accounting for a total caloric intake of 3000–3500 Kcals/day. On average, the trekkers spent 6–8 h walking daily.

**Table 1:** Daily diet during the Kilimanjaro Abruzzo Expedition

Breakfast	Water, tea, coffee, honey, muesli, banana, omelet, bread
Snack	Energy bars, banana, water
Lunch	Rice, eggs or omelet, chicken, vegetables, tea, coffee, water
Snack	Energy bars, banana, water
Dinner	Rice, eggs or omelet, chicken, vegetables, tea, coffee, water

### Anthropometric measurements

Body weight, height, body mass index (BMI), waist circumference and % body fat were recorded the day before the start of the mountain ascent (day 2) and the day after descending (day 8) in a laboratory of Moshi (Tanzania) at 900 m a.s.l. (Fig. 1). Skinfold data were obtained using a skinfold caliper and recorded to the nearest 0.2 mm and used to calculate % fat mass.

### Blood pressure, heart rate and blood oxygen saturation determination

Before (at day 0 and 2), during (at day 4, 5, 6 and 7) and after (at day 8 and 10) the trekking, arterial blood pressure (BP), Heart Rate (HR) and Oxygen Saturation (SpO_2_) were measured daily, in the morning, at rest (on awakening and fasting) (Fig. 1). BP and HR were recorded using an automatic electronic device (Omron M6, HEM-7001-E, Omron Healthcare, Hoofddorp, Netherlands), while blood oxygen saturation was evaluated by a finger pulse oximeter (503 OXY-5 GIMA, Gima S.p.A., Gessate, Italy). Participants were requested to rest in sitting position, without crossing their legs, and be silent before and during the measurements. Measurements were performed on the same arm in a horizontal position, by holding the arm at heart level, with a support bracket. Two blood pressures measurements (with 1-min interval) were averaged.

### Blood sample evaluation

In particular, 1 days before the trekking phase (day 2) and after reaching the summit of Mount Kilimanjaro, at the end of trekking (day 8), the volunteers were subjected to blood sampling, at 9 a.m., in a laboratory of Moshi (Tanzania, 900 m a.s.l.) (Fig. 1).

Post-expedition, blood specimens were collected and immediately cooled in ice, and then they were centrifuged at 3000×*g* for 10 min to isolate the serum. The sera were stored in liquid nitrogen and transferred to Italy to be analyzed in the Laboratory of Clinical Pathology, Hospital in Teramo.

Serum levels of glucose, creatinine, total cholesterol (T-Cholesterol), Low-Density Lipoprotein Cholesterol (C-LDL), High-Density Lipoprotein Cholesterol (C-HDL), triglycerides, aspartate aminotransferase/glutamic oxaloacetic transaminase (AST/GOT), alanine aminotransferase/glutamic pyruvic transaminase (ALT/GPT) and hemoglobin (Hb) were measured, along with hematocrit (Ht) and erythropoietin (EPO).

### Statistical analysis

All results were expressed as mean ± standard deviation (SD). Statistical pre–post comparisons were performed using the paired *t* test, whereas pressure, heart rate and oxygen saturation values were analyzed using the ANOVA for repeated measures and, when appropriate, the Newman–Keuls-based method. Differences were considered statistically significant with *P* < 0.05.

## Results

The anthropometric parameters of the twelve mountaineers (body weight, BMI and waist circumference), respectively, decreased (*P* ≤ 0.001), (*P* = 0.002) and (*P* = 0.032) but instead (body fat %) were not changed (*P* = 0.941) by the ascent to Kilimanjaro (see Table 2).

**Table 2:** Before (*departure from Moshi*) and after (*arrival in Moschi*) anthropometric characterization of twelve mountaineers enrolled in the Kilimanjaro Abruzzo Expedition

Anthropometry	Mean ± S.D	
	Before (departure from Moshi)	After (arrival in Moshi)
BW (Kg)	76 ± 10	74 ± 10***
BMI (Kg/m^2^)	26 ± 3	25 ± 3**
BF (%)	25 ± 3	25 ± 3
WC (cm)	90 ± 7	89 ± 7*

*BW* body weight, *BMI* body mass index, *BF* percentage of body fat and *WC* waist circumferences (means ± standard deviations)

* *P* < 0.05, ** *P* < 0.01, *** *P* < 0.001 vs before condition (paired *t* test)

The blood biochemical parameters, hemoglobin (Hb), hematocrit (Ht), erythropoietin (EPO), creatinine, glucose, aspartate transaminase/glutamic oxaloacetic transaminase (AST/GOT) and alanine transaminase/glutamic pyruvic transaminase (ALT/GPT) levels are reported as average ± standard deviation values in Table 3.

**Table 3:** Before (*departure from Moshi*) and after (*arrival in Moschi*) blood characterization of twelve mountaineers enrolled in the Kilimanjaro Abruzzo Expedition

Blood biochemical parameters	Mean ± S.D	
	Before (departure from Moshi)	After (arrival in Moshi)
Hb (g/dl)	15.3 ± 1.2	14.9 ± 1.3
Ht (%)	46 ± 3	46 ± 4
EPO (mIU/ml)	10 ± 4	8 ± 4
Creatinine (mg/dl)	1.05 ± 0.13	1.04 ± 0.16
Glucose (mg/dl)	89 ± 9	90 ± 4
AST/GOT (U/l)	24 ± 7	27 ± 7
ALT/GPT (U/l)	31 ± 11	32 ± 13

*Hb* blood content of hemoglobin, *Ht* hematocrit, *EPO* erythropoietin, *AST/GOT* creatinine, glucose, aspartate aminotransferase/glutamic oxaloacetic transaminase, and *ALT/GPT* alanine aminotransferase/glutamic pyruvic transaminase, was assessed

**Fig. 2:**
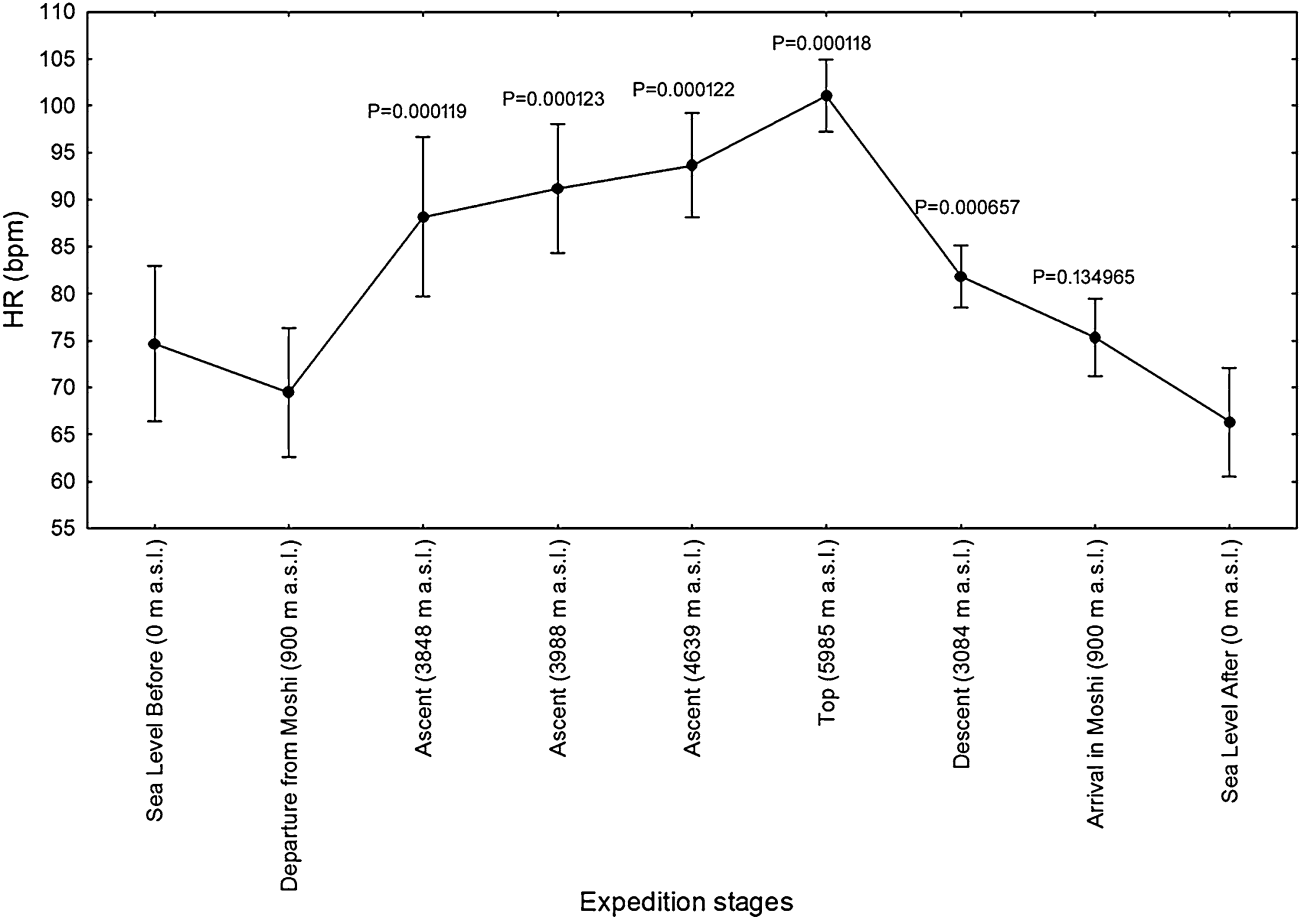
Heart rates (HR) of twelve mountaineers enrolled in the Kilimanjaro Abruzzo Expedition. Above 3000 m, HR were significantly higher, with respect to sea-level measurements. The main increase has been recorded on 5895 meters of altitude. *Error bars* 95 % confidence intervals. Newman–Keuls post hoc *P* values (vs. Departure from Moshi) are reported

**Fig. 3:**
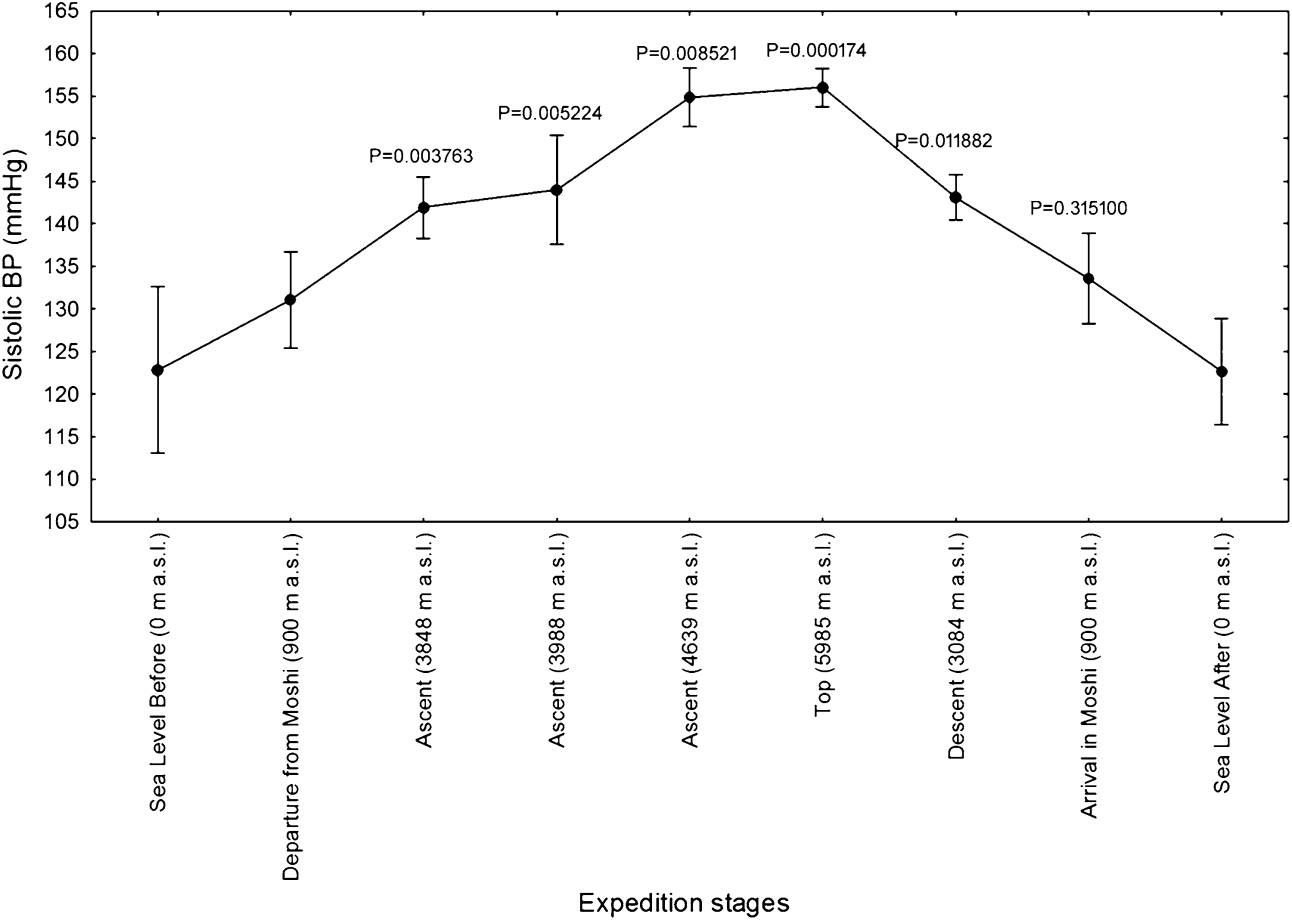
Systolic blood pressures (BP) of twelve mountaineers enrolled in the Kilimanjaro Abruzzo Expedition. Systolic BP progressively increased above 3000 m. The highest values have been recorded at altitudes over 4000 m a.s.l. *Error bars* 95 % confidence intervals. Newman–Keuls post hoc* P* values (vs. Departure from Moshi) are reported

**Fig. 4:**
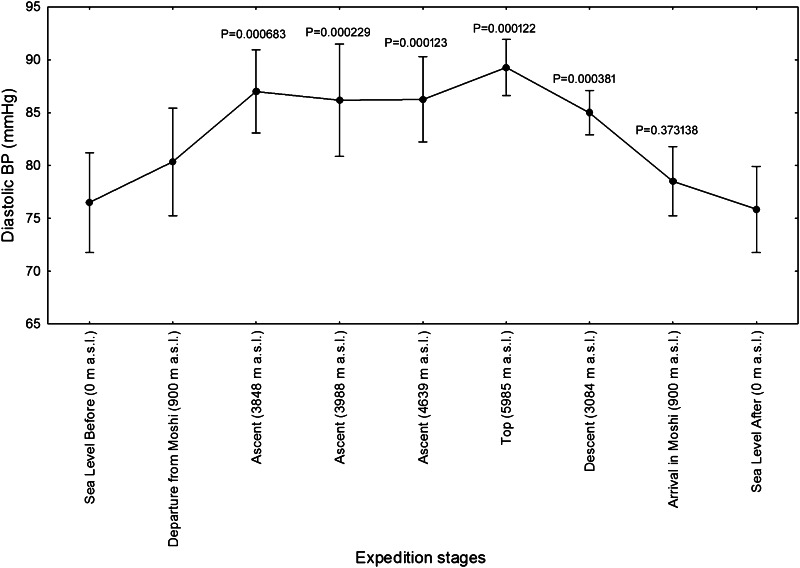
Diastolic blood pressures (BP) of twelve mountaineers enrolled in the Kilimanjaro Abruzzo Expedition. The highest values of the diastolic BP were progressively achieved over 3000 m a.s.l. *Error bars* 95 % confidence intervals. Newman–Keuls post hoc *P* values (vs. Departure from Moshi) are reported

**Fig. 5:**
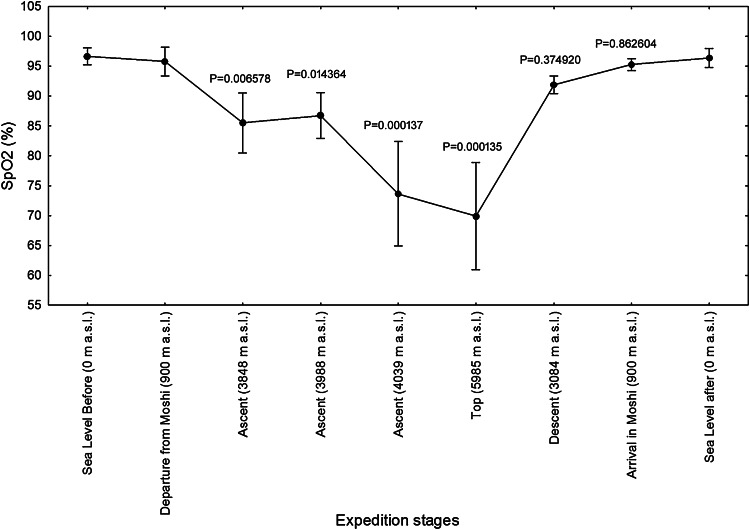
Blood oxygen saturation (SpO_2_) of twelve mountaineers enrolled in the Kilimanjaro Abruzzo Expedition. SpO_2_, considered as 100 % at sea level, decreased significantly above 3000 m of altitude, and the lowest values were observed above 4000. *Error bars* 95 % confidence intervals. Newman–Keuls post hoc *P* values (vs. Departure from Moshi) are reported

The cardiovascular parameters, heart rate (Fig. 2) and systolic and diastolic blood pressure (Figs. 3, 4), measured in the morning at rest, showed an increase gradually during ascent at high altitudes (*P* < 0.001), in comparison to s.l. conditions, while, as expected, during ascent blood oxygen saturation at altitude above 3840 m was found decreased when compared to s.l. (*P* < 0.001) (Fig. 5).

**Fig. 6:**
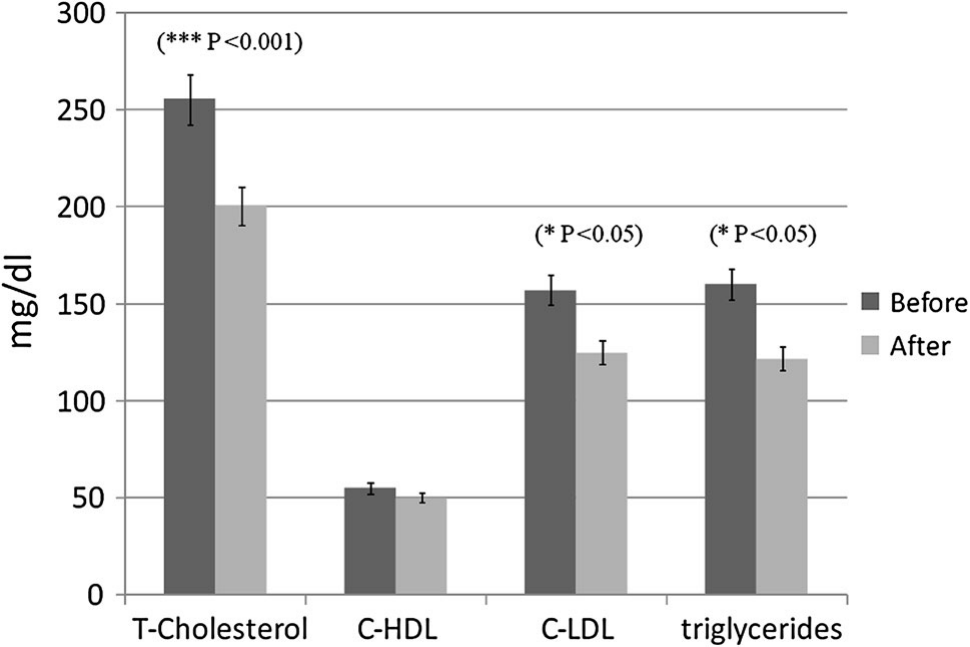
Pre- and post-lipid profiles of twelve mountaineers enrolled in the Kilimanjaro Abruzzo Expedition. Total cholesterol (T-cholesterol), C-LDL and triglycerides resulted significantly decreased immediately after the trekking experience

The high-altitude trekking significantly reduced triglycerides (*P* < 0.05) (Fig. 6), total cholesterol (*P* < 0.001) (Fig. 6) and C-LDL levels (*P* < 0.05) (Fig. 6), while C-HDL no significant changes (Fig. 6). Also, the ALT/GPT and AST/GOT ratios showed no significant changes.

Finally, hemoglobin, hematocrit, erythropoietin and creatinine levels were not affected by the expedition.

## Discussion

Trekking represents an optimal opportunity to study the effects of physical exercise performed at high altitude accompanied by a careful control of food and beverage supply and without possible effect of chronic adaptations. In this study, we analyzed the impact of a short trekking aimed to climb Kilimanjaro (Uhuru peak 5895 m a.s.l.) starting from Moshi at 900 m a.s.l. The ascent takes 6 full days while the way down takes only 2 days. Our attention was focused on anthropometric parameters, cardiovascular function and plasma lipid status with the perspective that physical exercise at altitude can improve health just acting on those specific parameters.

Although we did not detect major expedition-induced changes, probably due to the short duration of the altitude trekking and for the good control over food and beverage supply, our results showed slight, yet significant, changes in body composition, as on the other hand, it has been reported by other groups [15]. A healthy goal is to maintain a body weight that keeps the BMI score in the range of 25–20, which means fit-excellent categories. The Kilimanjaro trekking shifted BMI to fit category. It is worth mentioning that this shift does not provide additional health benefits “per se” but together with modified lipid profile is a good result.

Interestingly and surprisingly, in consideration of the short period of physical activity and high-altitude exposure, the trekking appeared to provide beneficial and rapid effects on the lipid profile. These effects probably rely on both high-altitude hypoxia and physical activity, as a consequence of hypoxia- and exercise-related adaptations [15, 16]. Actually, the effects of high-altitude exposure on plasma lipids and lipoprotein cholesterol have been previously studied. Férézou and colleagues found marked and persistent reductions in plasma cholesterol and phospholipids 3 days after arrival at 4800 m a.s.l. and the same author, also, found a decrease in plasma triglycerides 3 weeks after the high-altitude sojourn [17]. Low plasma cholesterol levels at the high altitude resulted mainly from the reduction in low-density lipoprotein cholesterol (C-LDL). It is worth noting that C-LDL variations were not related to body weight changes and that such modifications were not dependent on the activity during the expedition [17].

According to the study reported by de Mendoza, humans at high altitude exhibit significantly lower plasma total cholesterol and C-LDL levels, together with slightly lower amounts of high-density lipoprotein cholesterol (C-HDL), with respect to individuals living at low altitudes [18]. As shown by Domínguez Coello and colleagues, residents living at a higher altitude have increased C-HDL levels, compared to people living at sea level [19]. Similar results were reported in native populations living on high-altitude mountains [20, 21], as well as in a population migrating from lower altitudes to high mountain regions [22].

In the present work, we have also investigated the possible modulatory role of physical exercise at high altitude on inflammatory and metabolic pathways. Also, we have not found any difference on creatinine and transaminase levels at high altitude, as compared to sea-level assessments. These findings may exclude significant inflammatory responses in kidney and liver occurring during the expedition. On the other hand, we found a transient increase on blood pressure, which could be related, at least in part, to different mechanisms involved in BP regulation (i.e., neural central and reflex control of sympathetic activity), as recently reported by Parati et al. [23]. In strong agreement with previous works, we found a significant expedition-induced decrease in total cholesterol, C-LDL and triglyceride levels. This could suggest that the decrease of plasma C-LDL and triglyceride may reveal an adaptive response of the body to high-altitude hypoxic conditions [17].

Finally, we did not observe significant effects of high-altitude physical exercise on plasma glucose. Recent studies showed that physical exercise at moderate altitude could improve glycemic indexes, by affecting substrate utilization, thus outlining new prevention paradigms for metabolic and diabetes prevention [24, 25].

It was reported that high-altitude-induced activation of epinephrine-related pathways may improve both glycogenolysis and glucose usage [26] and this could, at least in part, explain the null effect on plasma glucose observed in mountain climbers who were exposed to moderate altitude for a short period [27].

The results obtained during Kilimanjaro expedition revealed a significant but temporary increase in arterial blood pressure at rest linked to the ascending and environment condition.

In conclusion, the analysis of health status, based on routine haematochemical measurements, determination of anthropometrical and cardiovascular parameters indicates that physical exercise, combined with a balanced diet, could activate positive adaptive mechanisms at high altitude. In particular, it is interesting to underline that high-altitude physical exercise, along with a balanced diet, ameliorates impaired lipid profiles.

## References

[1] Doria C, Toniolo L, Verratti V, Cancellara P, Pietrangelo T, Marconi V, Paoli A, Pogliaghi S, Fanò G, Reggiani C, Capelli C (2011). Improved VO_2_ uptake kinetics and shift in muscle fiber type in high-altitude trekkers. J Appl Physiol.

[2] Pelliccione F, Verratti V, D’Angeli A, Micillo A, Doria C, Pezzella A, Iacutone G, Francavilla F, Di Giulio C, Francavilla S (2011). Physical exercise at high altitude is associated with a testicular dysfunction leading to reduced sperm concentration but healthy sperm quality. Fertil Steril.

[3] Mariggiò MA, Falone S, Morabito C, Guarnieri S, Mirabilio A, Pilla R, Bucciarelli T, Verratti V, Amicarelli F (2010). Peripheral blood lymphocytes: a model for monitoring physiological adaptation to high altitude. High Alt Med Biol.

[4] Verratti V, Brunetti L, Tenaglia R, Chiavaroli A, Ferrante C, Leone S, Orlando G, Berardinelli F, Di Giulio C, Vacca M (2011). Physiological analysis of 8-ISO-PGF2 alpha: a homeostatic agent in superficial bladder cancer. J Biol Regul Homeost Agents.

[5] Bosco G, Verratti V, Fanò G (2010). Performances in extreme environments: effects of hyper/hypobarism and hypogravity on skeletal muscle. Myol Rev.

[6] Da Porto R, Trentini P, Verratti V, Petruccelli G, Di Giulio C (2009). Hypoxic ventilatory decline during the first 7 days of exposure in intermittent mountain altitude between 4400 and 6960 m. Sport Sci Health.

[7] Bogaard HJ, Hopkins SR, Yamaya Y (2002). Role of autonomic nervous system in the reduced maximal cardiac output at altitude. J Appl Physiol.

[8] Lhuissier FJ, Brumm M, Ramier D, Richalet JP (2012). Ventilatory and cardiac responses to hypoxia at submaximal exercise are independent of altitude and exercise intensity. J Appl Physiol.

[9] Naeije R (2010). Physiological adaptation of the cardiovascular system to high altitude. Prog Cardiovasc Dis.

[10] Wolfel EE, Selland MA, Mazzeo RS, Reeves JT (1994). Systemic hypertension at 4300 m is related to sympathoadrenal activity. J Appl Physiol.

[11] Hultgren HN (1992). Effect of high altitude on cardiovascular diseases. J Wilderness Med.

[12] Mazzeo RS, Child A, Butterfield GE, Mawson JT, Zamudio S, Moore LG (1998). Catecholamine response during 12 days of high-altitude exposure (4300 m) in women. J Appl Physiol.

[13] Van De Aardweg JG, Karemaker JM (1992). Repetitive apneas induce periodic hypertension in normal subjects through hypoxia. J Appl Physiol.

[14] Insalaco G, Romano S, Salvaggio A, Braghiroli A, Lanfranchi P, Patruno V, Donner CF, Bonsignore G (1996). Cardiovascular and ventilatory response to isocapnic hypoxia at sea level and at 5050 m. J Appl Physiol.

[15] Benso A, Broglio F, Aimaretti G, Lucatello B, Lanfranco F, Ghigo E, Grottoli S (2007). Enfdocrine and metabolic responses to extreme altitude and physical exercise in climbers. Eur J Endocrinol.

[16] Meckel Y, Nemet D, Bar-Sela S, Radom-Aizic S, Cooper DM, Sagiv M, Eliakim A (2011). Hormonal and inflammatory responses to different types of sprint interval training. J Strength Cond Res.

[17] Férézou J, Richalet JP, Coste T, Rathat C (1988). Changes in plasma lipids and lipoprotein cholesterol during a high altitude mountaineering expedition (4800 m). Eur J Appl Physiol Occup Physiol.

[18] de Mendoza S, Nucete H, Ineichen E, Salazar E, Zerpa A, Glueck CJ (1979). Lipids and lipoproteins in subjects at 1000 and 3500 meter altitudes. Arch Environ Health.

[19] Domínguez Coello S, Cabrera De León A, Bosa Ojeda F, Pérez Méndez LI, Díaz González L, Aguirre-Jaime AJ (2000). High density lipoprotein cholesterol increases with living altitude. Int J Epidemiol.

[20] Sharma S (1990). Clinical, biochemical, electrocardiographic and noninvasive hemodynamic assessment of cardiovascular status in natives at high to extreme altitudes (3000–5500 m) of the Himalayan region. Indian Heart J.

[21] Aitbaev KA (1985). The levels of high density lipoprotein and other lipids in the native population of the mountain region of Kirghizia (in Russian). Vopr Med Khim.

[22] Aitbaev KA, Madaminov I, Meimanaliev TS, Shleifer EZ, Kim NM (1990). Study of the effect of migration to high-mountain regions on the blood lipoprotein system (in Russian). Kosm Biol Aviakosm Med.

[23] Parati G, Ochoa JE, Torlasco C, Salvi P, Lombardi C, Bilo G (2015). Aging, high altitude, and blood pressure: a complex relationship. High Alt Med Biol.

[24] Haufe S, Wiesner S, Engeli S, Luft FC, Jordan J (2008). Influences of normobaric hypoxia training on metabolic risk markers in human subjects. Med Sci Sports Exerc.

[25] Lee WC, Chen JJ, Ho HY, Hou CW, Liang MP, Shen YW, Kuo CH (2003). Short-term altitude mountain living improves glycemic control. High Alt Med Biol.

[26] Kelly KR, Williamson DL, Fealy CE, Kriz DA, Krishnan RK, Huang H, Ahn J, Loomis JL, Kirwan JP (2010). Acute altitude-induced hypoxia suppresses plasma glucose and leptin in healthy humans. Metabolism.

[27] Katayama K, Goto K, Ishida K, Ogita F (2010). Substrate utilization during exercise and recovery at moderate altitude. Metabolism.

